# IRAK4 is an immunological checkpoint in neuropsychiatric systemic lupus erythematosus

**DOI:** 10.1038/s41598-024-63567-x

**Published:** 2024-07-16

**Authors:** Antoine Ménoret, Federica Agliano, Timofey A. Karginov, Xiangyou Hu, Anthony T. Vella

**Affiliations:** 1 Department of Immunology, UConn Health, 263 Farmington Ave, Farmington, CT 06030 USA; 2 Department of Neuroscience, UConn Health, 263 Farmington Ave, Farmington, CT 06030 USA

**Keywords:** Immunology, Autoimmunity, Innate immunity, Neuroimmunology

## Abstract

The search for dementia treatments, including treatments for neuropsychiatric lupus (NPSLE), has not yet uncovered useful therapeutic targets that mitigate underlying inflammation. Currently, NPSLE’s limited treatment options are often accompanied by severe toxicity. Blocking toll-like receptor (TLR) and IL-1 receptor signal transduction by inhibiting interleukin-1 receptor-associated kinase 4 (IRAK4) offers a new pathway for intervention. Using a pre-clinical NPSLE model, we compare lupus-like B6.MRL-Faslpr (MRL) mice with B6.MRL-Faslpr-IRAK4 kinase-dead (MRL-IRAK4-KD) mice, which are were less prone to ‘general’ lupus-like symptoms. We demonstrate that lupus-prone mice with a mutation in the kinase domain of IRAK4 no longer display typical lupus hallmarks such as splenomegaly, inflammation, production of hormones, and anti-double-stranded (ds)DNA antibody. water maze behavioral testing, which measures contextual associative learning, revealed that mice without functional IRAK4 displayed a recovery in memory acquisition deficits. RNA-seq approach revealed that cytokine and hormone signaling converge on the JAK/STAT pathways in the mouse hippocampus. Ultimately, the targets identified in this work may result in broad clinical value that can fill the significant scientific and therapeutic gaps precluding development of cures for dementia.

## Introduction

IRAK4 is a non-redundant signaling molecule responsible for relaying signals from IL-1R and TLRs (except for TLR3) to activate nuclear factor κB (NF-κB) and other important transcription factors, like CREB and AP1^1,2^, offering an alluring and practical pathway for intervention. IRAK4 operates through the assembly of the myddosome in which MyD88 recruits IRAK4 and the MyD88-IRAK4 complex recruits the IRAK4 substrates IRAK2 or the related IRAK1^3^. Formation of these complexes brings the kinase domains of IRAKs into proximity for phosphorylation and activation. Dysregulation of elements in the TLR signaling pathway is associated with many human immune system diseases like inflammatory disorders, autoimmune diseases, and allergy. Since TLRs have been implicated in SLE^4^, we and others suspected that blocking the non-redundant signaling of IRAK4 would limit SLE pathogenesis^5–10^.

SLE is an inflammatory autoimmune disease with a plethora of symptoms that most commonly include fatigue, joint pain, skin rashes, and fever. Its etiology is multifactorial and only partly understood. NPSLE is a manifestation of SLE generally impacting 40–60% of the SLE population, making NPSLE the least understood yet perhaps one of the most prevalent manifestations of lupus. NPSLE can affect the brain, spinal cord, and peripheral nerves, resulting in headaches, paresthesia, affective disorders, and cognitive dysfunction. However, despite this host of debilitating sequelae, NPSLE has an unspecified pathogenesis and limited treatment options, e.g., glucocorticoids and cyclophosphamide, which are only generically designed to treat inflammation and can be accompanied by severe toxicity^11^. The result is poor prognosis and high mortality, a dire situation demanding novel efforts to resolve the specific immune effectors of NPSLE and identify new therapeutic vistas. Though promising for SLE, IRAK4 inhibition has not yet been tested in NPSLE. However, IRAKs have been implicated in other neuroinflammatory diseases and neurodegenerative disorders with inflammatory involvement. For example, TLR4 and TLR2^12^, as well IRAK4^13^ have been implicated in the development of Alzheimer’s disease, while TLR2, TLR4, and TLR9 have been suggested to have a dominant role in Parkinson’s disease^14^.

Here, we report a kinetic study of lupus in MRL mice in which NPSLE symptomology is more common and progressive in females than males and thus consistent with sex differences observed in human. Using this model, we generated MRL-IRAK4-KD mice and analyzed the impact of IRAK4 mutation on splenomegaly, anti-dsDNA antibody, and cytokine and hormone production. We show a significant reduction of markers of disease in MRL-IRAK4-KD mice. By focusing on the hippocampus, we demonstrated a convergence of systemic cytokines and hormones toward the JAK/STAT pathway and showed this pathway is associated with memory deficiency as revealed by behavior testing relevant for NPSLE. RNA-seq data from SLE and NPSLE patients confirmed the clinical relevance of controlling the JAK/STAT pathway by blocking IRAK4.

## Results

### IRAK4 deficiency prevents hallmarks of lupus in mice predisposed to NPSLE

**Figure 1:**
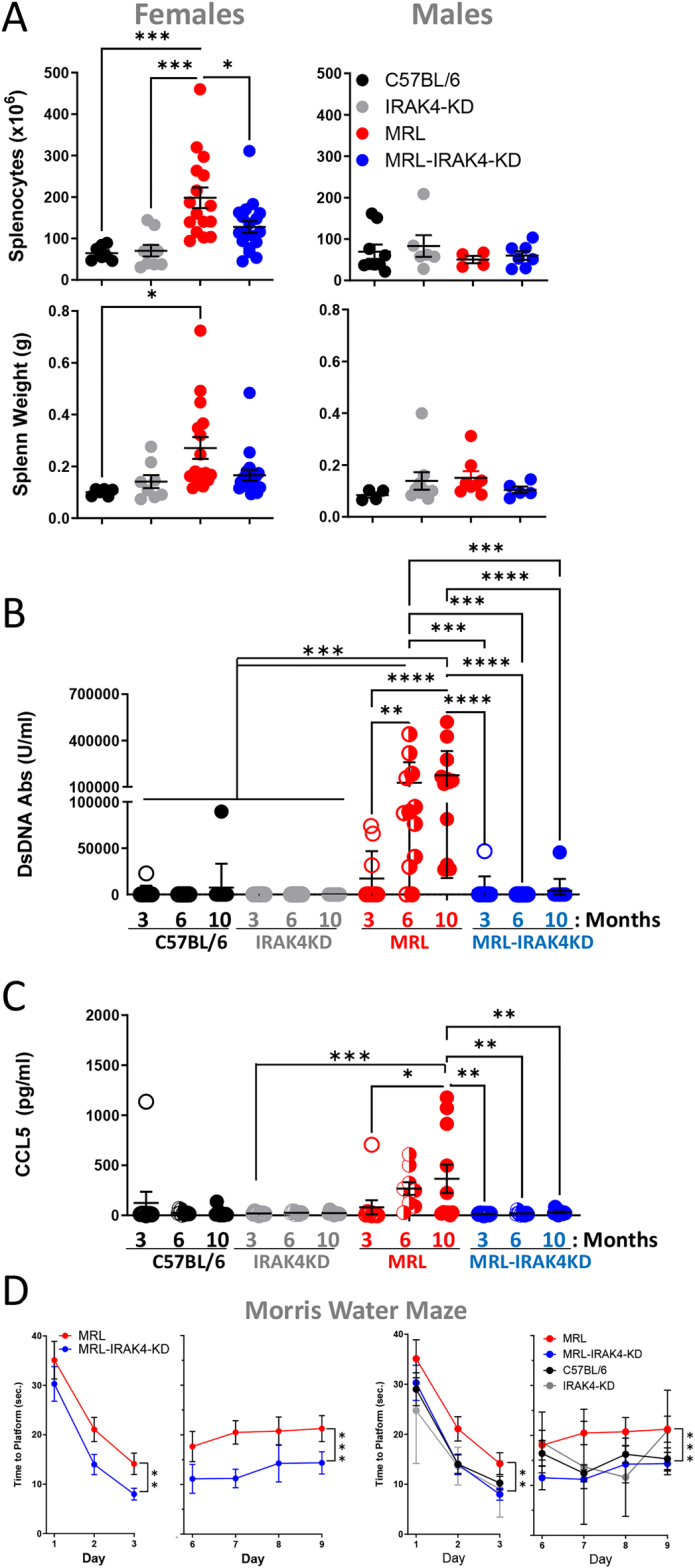
IRAK4 mutation abrogates NPSLE symptoms in MRL mice. (**A**) Splenocytes (top panels) and spleen weight (bottom panels) of 6 month-old female (left panels) and male (right panels) MRL, MRL-IRAK4-KD, C57BL/6, and IRAK4-KD mice were reported. Each dot represents one mouse. Sera were obtained from female MRL, MRL-IRAK4-KD, C57BL/6, and IRAK4-KD mice and quantified for (**B**) anti-dsDNA IgG antibodies and (**C**) CCL5 levels. Data are shown as mean +/− SEM. Each dot represents one mouse. *p < 0.05, **p < 0.01, ***p < 0.001, ****p < 0.0001 (one-way ANOVA). (**D**) Morris water maze (MWM) tests were applied to 10 month-old mice. Specifically, mice were tested for 3 consecutive days, rested for 2 days and tested daily for 4 additional days. *n* = 10–13/group. A mixed-effects model was used to compare differences in time-to-platform between the two groups after accounting for the repeated measures correlation^52^. We calculated overall mean difference between MRL and MRL-IRAK4-KD mice (two left panels) and 95% confidence interval (CI) during the observation period: days 1–3: on average, the difference was 6.05 s (95% CI 1.62–10.48) (*p* = 0.010). Days 6–9: on average, the difference was 7.26 s (95% CI 3.54–10.98) (*p* < 0.001). MWM analysis of all the mice strains is shown on the two right panels.

First, we tested if MRL mice display typical hallmarks of SLE and whether IRAK4 deficiency influenced these benchmarks. B6.MRL-Faslpr/J (MRL) mice were compared to MRL mice crossed with IRAK4-kinase-dead (MRL-IRAK4-KD) mice, both on C57BL/6 background. Splenomegaly was observed in female MRL mice but reduced in MRL-IRAK4-KD mice (Fig. 1A, left panels). Unlike females, males did not present with splenomegaly (Fig. 1A, right panels). These results recapitulated the more frequent manifestation of lupus in female patients and justify the use of female mice in further experiments^15^. Significantly, compared to MRL mice, MRL-IRAK4-KD mice also showed a profound reduction of anti-dsDNA antibodies (Fig. 1B), the presence of which has long been the gold standard of SLE^16^. Anti-dsDNA IgG antibodies in MRL mice were barely detected after 3 months of age, but their presence increased greatly by 6 months (Fig. 1B). This age-dependent progression of the disease in mice approximates the human condition. We previously showed that CCL5 is a marker of disease in a pristane-induced model of lupus^6^ where an IRAK4 inhibitor reduced splenomegaly and CCL5 secretion, implicating IRAK4 in control of CCL5 expression. Similarly, MRL-IRAK4-KD mice showed total inhibition of CCL5 secretion compared to MRL mice (Fig. 1C). Next, we investigated if these mice displayed symptoms specific to NPSLE and if these symptoms were alleviated by IRAK4 deficiency. To evaluate the impact of IRAK4 on known neuropsychiatric manifestations that typically develop late during SLE pathology, 10 month-old mice were subjected to a series of behavior tests. Spatial learning was evaluated by the Morris Water Maze (MWM), a protocol that measures the length of time it takes mice to navigate to a submerged escape platform located within an open swimming arena^17^. MWM demonstrated a clear statistical difference between strains. After 3 days’ testing, MRL-IRAK4-KD mice showed stronger spatial learning compared to MRL mice as evidenced by a significantly lower time-to-platform measurement; this difference was maintained through 9 days of testing (Fig. 1D, two leftmost panels). MRL-IRAK4-KD mice behavior was comparable with IRAK4-KD and C57BL/6 control mice (Fig. 1D, two rightmost panels). Other behavior tests revealed equivalent anxiety, locomotor, and exploratory activity in MRL and MRL-IRAK4-KD mice indicating similar neuromuscular potentials (Suppl. Fig. 1A, B). Fear conditioning behavior test was also used as an additional amygdala-dependent associative learning measure but showed no differences between MRL and MRL-IRAK4-KD mice (Suppl. Fig. 1C). All together, these behavioral tests support our hypothesis that IRAK4 activity is critical for NPSLE development, and specific for contextual associative learning defects associated with the hippocampus^17^. These data suggest that IRAK4 activity is critical for NPSLE development and especially contextual associative learning defects.

### IRAK4 deficient mice have a lower potential for innate response but are not immunocompromised

**Figure 2:**
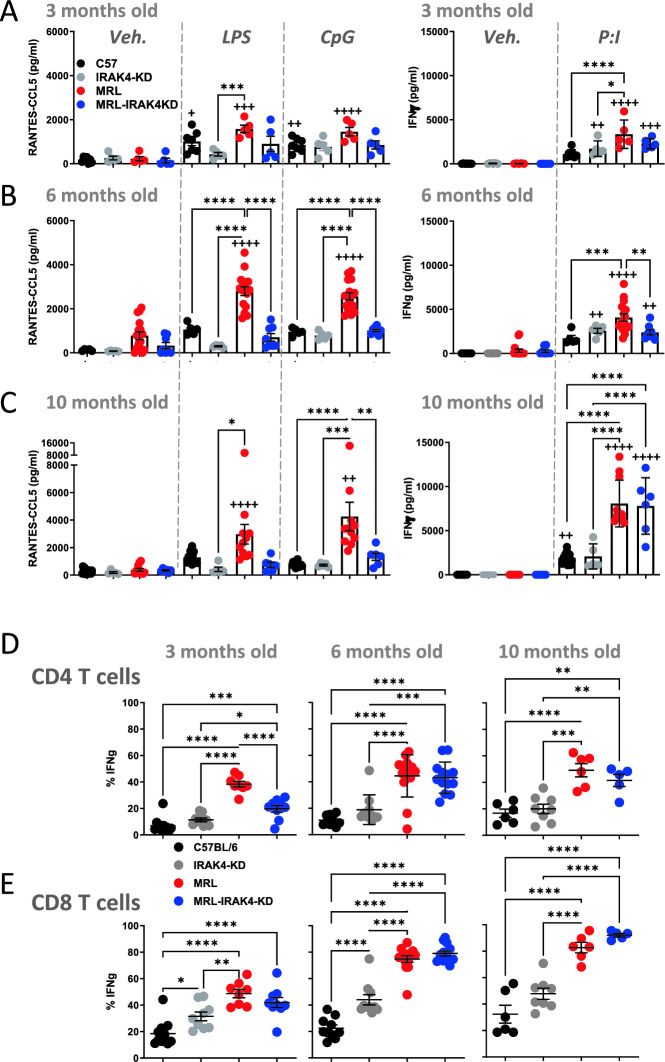
IRAK4 mutation impacts innate and adaptive cytokine response in MRL mice. Splenocytes from 3 (**A**), 6 (**B**), and 10 month old mice (**C**) were simulated with vehicle (Veh.), *E. coli* LPS (100 ng/ml), CpG (1 µM) for 18 h (left panels) or with PMA and ionomycin (P:I) for 18 h (right panels). Culture supernatants were obtained at 18 h and assayed for RANTES/-CCL5 (left panels) and (IFNγ) by ELISA. Splenocytes from 3, 6, and 10 month old mice were stimulated with PMA and ionomycin (PMA:I) for 5 h in the presence of Brefeldin A (BFA). Cells were analyzed by flow cytometry, Percentage of intracellular IFNγ expressing cells is shown for CD4 T cells (**D**) identified as live CD3^+^, CD4^+^ and CD8 T cells (**E**) identified as live, CD3^+^, CD8^+^. Each dot represents a mouse. Data shown are individual biological replicates +/− s.e.m. Statistical significance was evaluated by one way ANOVA, *p < 0.05, **p < 0.01, ***p < 0.001, ****p < 0.0001 for comparison of individual biological within a treatment group and ^+^*p* < 0.05, ^++^*p* < 0.01, ^+++^*p* < 0.001, ^++++^*p* < 0.0001 for comparison of individual biological with the same mouse train in vehicle treated group.

To assess the potential of mouse splenocytes to respond to innate stimuli we stimulated splenocytes with LPS and CpG and measured CCL5 release (Fig. 2A–C, left panels). MRL mice showed an enhanced potential for CCL5 release especially at 6 and 10 months that is abrogated in MRL-IRAK4-KD splenocytes (Fig. 2B, C, left panels). Interestingly secretion of IFNγ from splenocytes stimulated with phorbol 12-myristate 13-acetate and ionomycin (PMA:I) increased with the age of the mouse and was more pronounced in the MRL background mice, but no difference was observed between MRL and MRL-IRAK4-KD strains (Fig. 2A–C, right panels). To determine the origin of IFNγ, we performed intracellular cytokine staining in splenocytes stimulated for 5 h with PMA:I and again observed an increased potential for IFNγ synthesis with the age of the mouse, especially for CD8 T cells (Fig. 2D, E). Generaly, MRL and MRL-IRAK4 CD4 and CD8 T cells had a general higher potential to synthesized IFNγ compared to T lymphocytes from C57/BL6 and IRAK4-KD mice. No difference in IFNγ expression was observed between MRL and MRL-IRAK4 CD4 and CD8 T cells except in CD4 T cells from 3 month old mice (Fig. 2D, left panel). Together, these data demonstrate an attenuation of the innate potential of MRL-IRAK4-KD splenocytes, but not adaptive lymphocytes, when compared to lymphocytes from MRL mice.

### IRAK4 deficiency has negligeable influence on the microbiome but regulates lupus-associated hormones

While we demonstrated that IRAK4 influences NPSLE manifestation, the mechanism underlying IRAK4’s action was unclear. Thus, we wanted to investigate several means by which IRAK4 deficiency alleviated NPSLE symptoms. The microbiome is a source of pathogen-associated molecular patterns (PAMPs) that may initiate immune responses. Several TLRs convey the presence of PAMPs through IRAK4 signaling; thus, IRAK4 depletion may impact subsequent immune response by preventing PAMP detection. However, it remains possible that the microbiomes between MRL, MRL-IRAK4-KD are different, and control mice, making it difficult to pinpoint IRAK4 deficiency as the sole driver of diminished pathology in MRL-IRAK4-KD mice. To resolve this, our group compared microbiomes from MRL and MRL-IRAK4-KD mice. The 50 most abundant bacterial strains detected between 3 and 10 months were found to be nearly identical between treatments (Suppl. Fig. 2A), except for one single bacterium (*Parasutterella excrementihominis*) whose absence in MRL mice is both IRAK4-dependent and whose presence is inversely associated with symptoms (Suppl. Fig. 2B). Considering the low frequency of this bacterium in control mice (~ 0.001% of total), these results strongly suggest that the overall microbiome composition is neither IRAK4-dependent nor the trigger for the increase of IRAK4-dependent pathology observed in MRL mice.

**Figure 3:**
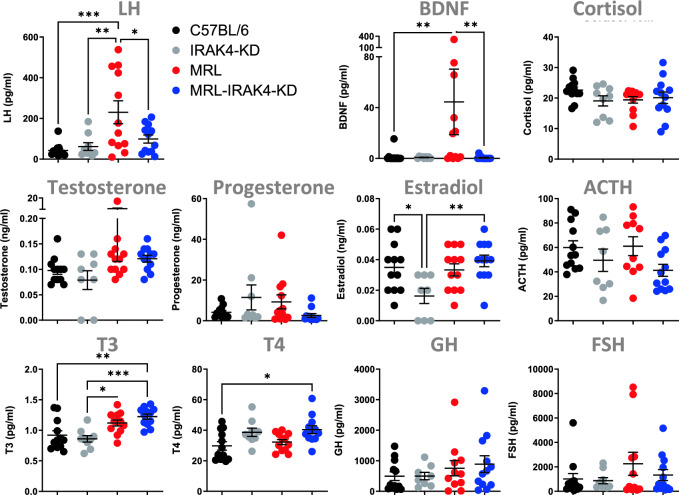
IRAK-4-dependent lupus-associated hormones. Sera were obtained from 10 month-old female MRL, MRL-IRAK4-KD, C57BL/6, and IRAK4-KD mice and quantified. Data are shown as mean +/− SEM. *p < 0.05, **p < 0.01, ***p < 0.001 (one-way ANOVA).

Since the influence of the differences in the microbiomes seemed minimal, we considered the changes in hormonal levels relevant for NPSLE pathology and associated with onset and flares^18^. We investigated hormone differential expression between MRL mice with fully developed pathology (10 month-old) and age-matched MRL-IRAK4-KD mice. Luteinizing hormone (LH) and brain derived-neurotrophic factor (BDNF) were upregulated in MRL mice and low in MRL-IRAK4-KD and control strains (Fig. 3, two top left panels), whereas levels of the other hormones tested were similar across mice strains (Fig. 3). These observations appeared to depend on the extent of the pathology, with differences in LH and BDNF not detected at 3 month (Suppl. Fig. 3B), but started to manifest by 6 months (Suppl. Fig. 3B) and were more pronounced at 10 month (Fig. 3, two top left panels). Thus, our results reveal that key hormone differences in LH and BDNF but not the microbiome may function in NPSLE pathology. Overproduction of LH, a hormone that is critical in ovulation and pregnancy, could be responsible for the decrease of mouse progeny in MRL mice compared to MRL-IRAK4-KD (Suppl. Fig. 3C). Interestingly, expression of both LH and BDNF are known to be increased in SLE patients and are also known to participate in cognitive decline through loss of memory most likely associated with the hippocampus^19–22^. Extending this reasoning, our group tested the hypothesis that IRAK4 is an immunological checkpoint and a relevant therapeutic target in controlling neuropsychiatric lupus (NPSLE) and especially the hippocampus as a center for memory acquisition and behavior.

### IRAK4 controls JAK-STAT and cytokine/chemokine-dependent pathways in the hippocampus of NPSLE-prone mice

**Figure 4:**
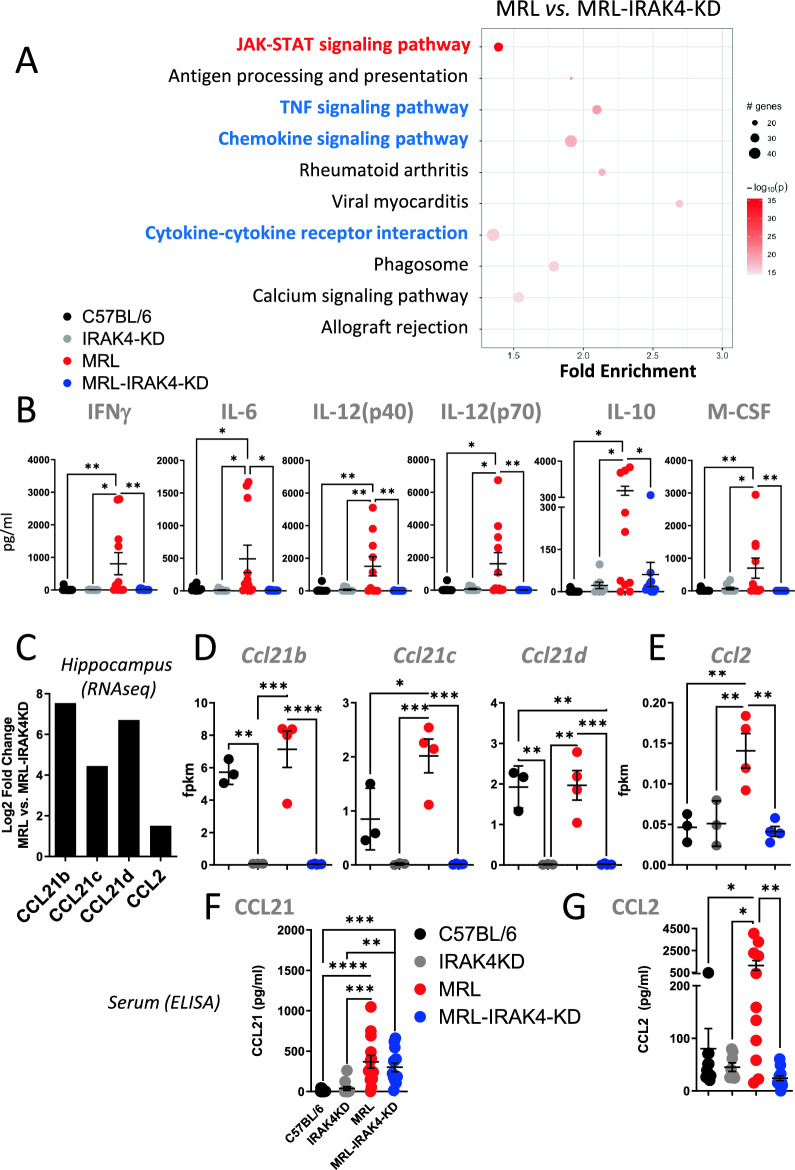
IRAK4 increases hippocampal Jak-STAT pathway in hippocampus of lupus-prone mice. (**A**) RNA was extracted from 10 month-old MRL and MRL-IRAK4-KD hippocampi after brain perfusion and sequenced by RNA-seq (Whitehead Institute). 3114 filtered genes with *p* value < 0.05 between MRL and MRL-IRAK4-KD groups (*n* = 4/group) were used for subsequent pathway analysis using pathfindR. Fold enrichment was reported for each pathway. (**B**) Cytokine levels of sera from 10 month-old female MRL, MRL-IRAK4-KD, C57BL/6, and IRAK4-KD mice. Data are shown as mean +/− SEM. Each dot represents one mouse. *p < 0.05, **p < 0.01 (one-way ANOVA). (**C**) Log_2_ fold change difference of all chemokines detected by RNA-seq [from (**A**)] between MRL and MRL-IRAK4-KD were analyzed; the most significant are shown. (**D**) Individual mRNA Ccl21 isoforms (b, c, and d), (**E**) Ccl2 mRNA expression in the hippocampus, (**F**) CCL21 and (**G**) CCL2 levels of sera from 10 month-old MRL, MRL-IRAK4-KD and control mice were measured by ELISA. Each dot represents one mouse. Data are shown as mean + / − SEM. *p < 0.05, **p < 0.01, ***p < 0.001, ****p < 0.0001 (one-way ANOVA).

LH and BDNF, importantly, are known to participate in cognitive decline through loss of memory most likely associated with the hippocampus^19–21^. Thus, we sought to investigate changes in the hippocampus via RNA-seq. We performed RNA-seq on hippocampi from MRL vs. MRL-IRAK4-KD mice. Pathway analysis showed an enrichment in JAK-STAT, TNF, and chemokine signaling pathways in MRL over MRL-IRAK4-KD mice (Fig. 4A). The JAK/STAT pathway was not the most enriched pathway but by far the most statistically significant enriched pathway (Suppl. Fig. 6). The enrichment in these signaling pathways could reflect actions of systemic circulating or locally produced cytokines. Circulating JAK/STAT-dependent cytokines IFNγ, IL-6, IL-12(p40), IL-12(p70), IL-10, and M-CSF were specifically detected in MRL mice serum, but not in control mice (Fig. 4B), whereas RNA-seq of the hippocampus demonstrated either absence, very low counts or no difference in mRNA transcripts of the same JAK/STAT-dependent cytokines (Supp. Figure 4A). Enrichment in JAK/STAT pathway may also depend on increased systemic BDNF and LH (Fig. 3) that can both signal through that pathway^23,24^ and, like for the cytokines, their transcripts are not differentially expressed in the hippocampus (Supp. Figure 4B). This data suggests the pathway enrichment was via systemic circulating JAK-STAT-dependent cytokines and hormones but not due to their synthesis by the hippocampus. Moreover, the enrichment in chemokine signaling pathways is congruent with the notion that the hippocampus is a wellspring of chemokines, some of which have been suggested to contribute to NPSLE^25^. Hence, the RNA-seq data was mined for chemokines matching the enrichment of the chemokine pathway. We found CCL21 isoforms b, c, and d and CCL2 (MCP-1) chemokines to match the chemokine pathway enrichment, which showed a strong statistically significant increase in MRL mice compared to MRL-IRAK4-KD mice (Fig. 4C). Although CCL21 mRNA expression depended on IRAK4, its expression was not as clearly associated with NPSLE as its mRNA was also present in the hippocampi of C57BL/6 mice (Fig. 4D, third column in each CCL21 plot). Moreover, expression of CCL21 protein in serum was similar between MRL and MRL-IRAK4-KD mice (Fig. 4F). In stark contrast, both mRNA and protein expression of CCL2 were increased only in MRL mice (Fig. 4E, G). Based on these data, we hypothesized the activation of these pathway in the hippocampus would manifest in NPSLE-related behavior. Here, our data reveals an upregulation of the JAK/STAT pathway via systemic circulating cytokines and an upregulation of CCL2 in the hippocampus of MRL mice, both of which were abrogated by IRAK4 deficiency concurrent with a reduction in NPSLE symptoms. These results provided potential targets for intervention.

## Discussion

Our study provides evidence that mice susceptible to NPSLE with mutated IRAK4 kinase domain improved their pathology, including reduced splenomegaly, absence of anti-dsDNA antibody and better memory function congruent with hippocampal function (Fig. 1). One advantage of the MRL (B6.MRL-Faslpr) mice for studying spontaneous SLE and NPSLE is that these mice exhibit progressive development of the disease through several months. However, one limitation of MRL mice compared to other models of spontaneous SLE is that they develop extreme lymphadenopathy^26^. This condition is rarely present in human patients. Although loss of Fas or Fas-L ligand are rarely present in SLE patients^27^ and can be seen as an apparent limitation of the MRL model, Fas and Fas ligand gene polymorphism is associated with risk of developing SLE^28,29^. In our study, pathological signs appear only in females and after 6 months, suggesting a cumulative effect of IRAK4 deficiency possibly linked with TLR stimulation from microbe-derived PAMPs, but this hypothesis was not supported based on the minimal differences observed between the mouse strains (Suppl. Fig. 2). On the contrary, the progression of disease was observed as there was an increase in systemic hormones (Fig. 3) and cytokines (Fig. 4B) that stimulate the JAK/STAT pathway, a pathway specifically enhanced in mouse hippocampus (Fig. 4A).

The influence of PAMPs in other SLE models has been suggested by the effect of antibiotic treatment given after disease onset. This treatment removes bacteria from the gut microbiota and further attenuates SLE-like disease^30^. However, in the present model with intact microbiome, the longitudinal time study from MRL, MRL-IRAK4-KD, and control mice showed no pattern of bacterial expression associated with disease development (Suppl. Fig. 2A) with the exception of one single bacterium (*Parasutterella excrementihominis*) which is absent in MRL mice, IRAK4-dependent, and inversely associated with NPSLE symptoms (Suppl. Fig. 2B). However, because this newly characterized bacterium^31^ is absent in MRL and extremely unabundant in the other mouse strains (~ 0.001% of total), we favor the hypothesis that soluble TLR ligands released from dead cells (such as cell-free DNA, RNA, or HMGB1, etc.), could trigger systemic inflammation rather than differences in gut microbiota^32^. This supports our previous observation that an IRAK4 inhibitor can block pristane-induced lupus-like inflammation in presence of high concentration of serum cell-free DNA^6^, although in the current study cell-free DNA level in MRL mouse sera was low and not different from control mice (Suppl. Fig. 5).

The molecular analysis of the hippocampus revealed enrichment in pathways all involved in the immune response (Fig. 4A). Interestingly, enrichment of JAK/STAT was coincident with circulating BDNF and LH both of which can signal through this pathway^23,24^ and are involved in learning and memory processes located in the hippocampus^19,21^, but are not expressed differentially in the hippocampus (Suppl. Figure 4B). Moreover, six circulating cytokines known to signal through the JAK/STAT pathway were highly expressed in MRL mice (Fig. 4B). Other murine models of NPSLE have shown increased of cytokines (IL-6, IL-10, IL-12, IL-18 and, IFNγ) in the hippocampus that could induce JAK-STAT signaling^33–35^. The mechanism by which IRAK4 affects the JAK/STAT pathway has not been studied in NPSLE but is reminiscent of the effect of an IRAK4 competitive inhibitor that has been shown to decrease NF-κB and STAT3 signaling, cytokine secretion, and proliferation^36^.

Our data argue for a convergence of circulating hormones and cytokines toward a common JAK/STAT pathway to directly stimulate cells of the hippocampus. This might be exemplified by CCL2 (Fig. 4C–G), whose expression is not known to be IRAK4-but STAT6-dependent^37^. CCL2 has been implicated in several neuroinflammatory pathologies through its effect on microglia^38^ via its receptor, CCR2, leading to the production of pro-inflammatory cytokines and chemokines such as TNF-α, IL-1β, and IL-6. Moreover, the presence of CCL2 in the CSF of NPSLE patients reflects an inflammatory activity in the brain and suggests it could attract immune cells to the site^39^. To date, however, the role of and mechanism by which CCL2 acts in NPSLE has not been explored. However, a limitation of this study is the global deficiency in IRAK4 kinase activity in MRL mice. A cell specific IRAK4-KD model would help test which cell population is responsible for the improvement in spatial memory.

JAK/STAT inhibitors for NPSLE treatment have been suggested elsewhere^40^. These inhibitors interfere with JAK-STAT signaling, they are small molecules that penetrate the BBB^41^, and reduce the production of several cytokines. Tofacitinib, a JAK1/JAK3 inhibitor, is currently in phase II studies for SLE treatment and is worthy of consideration in this regard^42^. Hence, JAK-STAT inhibitors have been shown initial promising results for SLE treatment although with limited serological activity^43^. However, two recent phase 3 clinical trials using a selective JAK1/2 inhibitor have been discontinued after disappointing results^44,45^. Critically, in these two trials, exclusion criteria prohibited recruitment of NPSLE patients, a sub-category of SLE patients that could benefit from a JAK inhibitor. Our study suggests that future trials testing a JAK inhibitor would be more successful with the specific inclusion of NPSLE patients.

Considering the limited progress of using JAK inhibitor in the clinic, blocking IRAK4 upstream of this pathway offers the possibility for blocking at once TNF and JAK/STAT pathways along with alleviating anti-dsDNA load using only one inhibitor, an approach that may better control SLE and NPSLE.

Altogether, our study sheds new light on a novel link between inflammation and memory loss, providing a systemic mechanism that underscores the importance of targeting IRAK4, a checkpoint for neuroinflammation, for controlling hormones and cytokines that affect the hippocampus and the behavior during NPSLE.

## Methods

### Mice

C57BL/6 mice were purchased from the Jackson laboratory (Bar Harbor, ME). B6.MRL-Faslpr/J (MRL) mice, MRL mice crossed with IRAK4-kinase-dead (MRL-IRAK4-KD) mice, IRAK4-kinase-dead (IRAK4-KD) mice, all on C57BL/6 background were obtained from Dr. Andrei Medvedev (UConn Health). All the mice used in the study were euthanized after completion of the experiments in a carbon dioxide chamber. All mice were maintained in the central animal facility at UConn Health in accordance with federal guidelines. The present study was approved by the UCH’s animal care committee. The study is reported in accordance with ARRIVE guidelines.

### Tissue processing

For hippocampus isolation, mice were perfused with cold PBS before hippocampus dissection. For RNA analysis, hippocampi were lysed in trizol lysis buffer. RNA was extracted and processed using a QIAGEN miRNEasy extraction kit. For protein analysis, hippocampi were lyzed in hippocampus were homogenized in 20 mM Tris, 150 mM NaCl, 1 mM EDTA, 2 mM EGTA, 2 mM glycophosphate, 10 mM NaF, 1 mM orthovanadate, 1 µg/ml leupetine. A 25,000 g supernatant was obtained, and protein was quantified using BCA assay (Thermo Fisher Scientific, Waltham, MA). For splenocyte isolation, spleens were crushed through nylon mesh cell strainers (Falcon/BD Biosciences, San Jose, CA, USA) and treated with ammonium chloride to lyse RBCs.

### Multiplex and ELISA and multiplex

CCL5 and CCL21 Elisa kits were purchased from BD bioscience (San Jose, CA). Multiplex for cytokines/chemokines and hormones detection were purchased from Millipore (Burlington, MA). Anti-ds DNA kit was purchased from Alpha Diagnostic Intl. Inc (San Antonio, TX).

### Flow cytometry

Flow cytometry was performed as described before^46^. Cells were incubated for the last 5 h in the presence of GolgiPlug (BD Biosciences), stimulated with phorbol 12-myristate 13-acetate (PMA) purchased from EMD Millipore Corporation (Billerica, MA) and ionomycin purchased from Life Technology (Grand Island, NY). Cells were stained with live/dead UV blue stain purchased from InVitrogen (San Diego, CA) and surface stained with anti-CD3 antibody (BD bioscience, catalog#557984), anti-CD4 antibody (BD bioscience, catalog#552051) and anti-CD8 antibody ebioscience, catalog#48-0081-82), fixed with 1.5% PFA, permeabilized with 1% saponin, and stained at 4 °C overnight with IFNγ (BD PharMingen, catalog# 554412, Clone XMG1.2, Franklin Lake, NJ). Acquisition was performed by LSRIIa. All flow cytometry data were analyzed with FlowJo (Tree Star, Ashland, OR).

### RNA seq

#### Bulk RNA-seq

For each group, hippocampi were lysed in trizol lysis buffer. RNA was extracted and processed using a QIAGEN miRNEasy extraction kit. RNA library construction was done using a SMART-seq v.4 ultra-low input kit (Takara Bio, Shiga, Japan) and cDNA was converted to sequencing library using NexteraXT DNA library prep kit with indexing primers (Illumina, San Diego, CA). For transcriptomics, libraries were sequenced for single end 1 × 100 bp reads at 30 million reads/sample on a NOVASeq 6000 (Illumina). Quality controlled reads (fastq) were aligned to mouse genome (mm10) using HISAT2 and BAM file conversion, sorting, and indexing were done with Samtools^47,48^. Read counts were obtained from resulting BAM files using Stringtie^49^. For transcriptomics analysis, differentially expressed genes were identified using DESeq2 (p-value < 0.05)^50^. Pathway analysis was conducted using PathfindR^51^. Library preparation and sequencing was conducted by the Whitehead Institute Genome Technology Core (Boston, MA).

### Behavior tests

Water maze was used to study spatial learning and memory. The tank is filled with 20–22 °C water for a 9 day test. In the first 3 days, the mice are put into the tank with water from four different directions. The mice are tested for 60 s each time to locate a circular platform placed in the center of one of the four quadrants of the pool so that the top is around 0.5 cm above the water surface with a red visual cue. The time to reach the platform will be measured by video AnyMaze software. In the following 4 days, the platform is kept in the same position, but the platform top is 0.5 cm below the water surface and red visual cue were removed from the platform.

Open Field was performed by placing mice in an open field arena for 10 min and their free movement is recorded via video AnyMaze software. Exploratory activity, anxiety, and hyperactivity were evaluated by analyzing total moving distance, average speed, center time & distance, corner time and distance.

Y-Maze was used to measure spontaneous alternation has a behavioral test for measuring the willingness of rodents to explore new environments. For this test, mice were put into one arm of the Y-maze and allowed to freely explore the three arms for 5 min to assess the spontaneous alterations.

Fear conditioning is measured to assess cognitive function in mouse. The standard test needs 3 days. On the first day, which is the conditioning period, the mice will be placed in the conditioning chamber for 3 min before onset of a 30 s sound stimulus at 2800 Hz and 85 dB. The last 2 s of the sound stimulus will be coupled with a foot shock (0.5 mA of continuous current). After resting an additional 30 s in the chamber, the mice will be repeated the sound and foot shock again, and returned to home cage. On the second day, the mice will be tested for contextual memory in the same chamber for 3 min without either sound or foot shock. On the third day, the mice will be tested for tone memory in the same chamber with different environment. The mice will only suffer the sound stimulus, but no foot shock. Fear memory is measured as the percentage of freezing, which is defined as the percentage of time completely lacking movement in intervals of 5 s. In between animals, the instrument will be completely cleaned with 70% ethanol to remove the odor and dirty materials^17^.

### Statistical analysis

Two-tailed student’s unpaired and one-way Anova tests used for data analysis, with values of *p* < 0.05 (*) used as significant threshold; *p* < 0.01 is indicated as (**), *p* < 0.001 as (***) and *p* < 0.0001 is indicated as (****) were performed using Prism-GraphPad (La Jolla, CA).

### Supplementary Information

Supplementary Figures.

## Data Availability

The datasets generated and analyzed during the current study are available in the Gene Expression Omnibus database “GEO” under the repository accession number GSE264121.
