# Supplementary material for: IRAK4 is an immunological checkpoint in neuropsychiatric systemic lupus erythematosus
Source: Sci Rep. 2024 Jul 16;14:16393. doi: 10.1038/s41598-024-63567-x (PMC11252422; doi:10.1038/s41598-024-63567-x)
Supplement: Supplementary file 1 — Supplementary Figures. [file 41598_2024_63567_MOESM1_ESM.docx]

**Supplementary Figure legends**

**Supplementary Figure 1. IRAK4 deficiency has negligeable influence on the microbiome. A.** Microbiomes contained in feces of MRL, MRL-IRAK4-KD, C57BL/6, IRAK4-KD mice were identified by sequencing (Diversigen) and reported as relative abundance of bacteroid species in 3-, 6- and 10-month-old mice. **B**. Multiple statistical comparison for *Parasutterella excrementihomi* frequency among mice stains is reported.

**Supplementary Figure 2.** **Behavior tests in MRL and** **MRL-IRAK4-KD mice.** Behavior tests were applied to 10-month-old mice. Mice were run through **A.** Open Field, **B.** Y Maze, and **C.** Fear Conditioning. *n*=10–13/group. Each dot represents a mouse.

**Supplementary Figure 3. IRAK-4-dependent lupus-associated hormones.** Sera were obtained from **A.** 3-month-old and **B.** 6-month-old female MRL, MRL-IRAK4-KD, C57BL/6, and IRAK4-KD mice and quantified. Data are shown as mean +/- SEM. **p* < 0.05, ***p* < 0.01, ****p* < 0.001 (one-way ANOVA). **C.** Number of pups born from female 3–8-month-old mice. Each dot represents one mouse. Data are shown as mean +/- SEM. ***p* < 0.01 (Student’s *t*-test).

**Supplementary Figure 4. IRAK4 does not control RNA expression of JAK/STAT-dependent hormones and cytokines in hippocampus of NPSLE-prone mice.** **A.** RNA of BDNF and LH and **B.** RNA of all interleukins, M-CSF and IFNγ from 10-month-old MRL, MRL-IRAK4-KD, C57B/L6, and IRAK4-KD extracted mouse hippocampi as described in Fig. 3 are shown as mean +/- SEM. Each dot represents one mouse.

**Supplementary Figure 5. IRAK4 does not alter cfDNA release in the serum of NPSLE-prone mice**. Sera were obtained from 6-month-old female MRL, MRL-IRAK4-KD, C57BL/6, and IRAK4-KD mice were quantified for level of cfDNA (cell-free DNA). Each dot represents one mouse (n = 10-12 mice per group).

**Supplementary Figure 6. Fold enrichment of pathways in hippocampus of lupus-prone mice.** RNA was extracted and analyzed as described in legend of Fig. 4. Pathways described in Fig. 4 were ranked by order of fold enrichment (Y axis) in context of -log of p adjusted value (X axis).

**
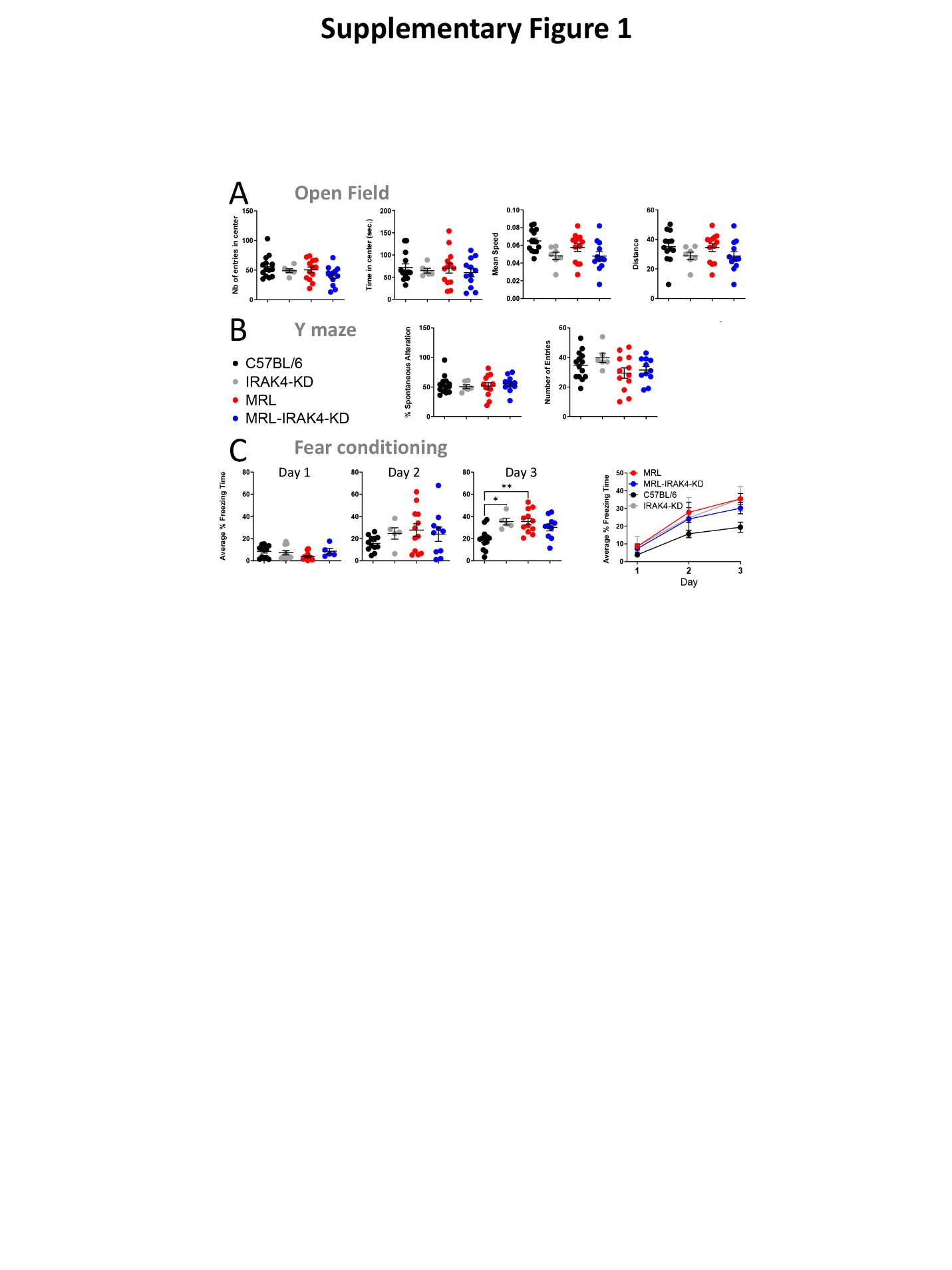

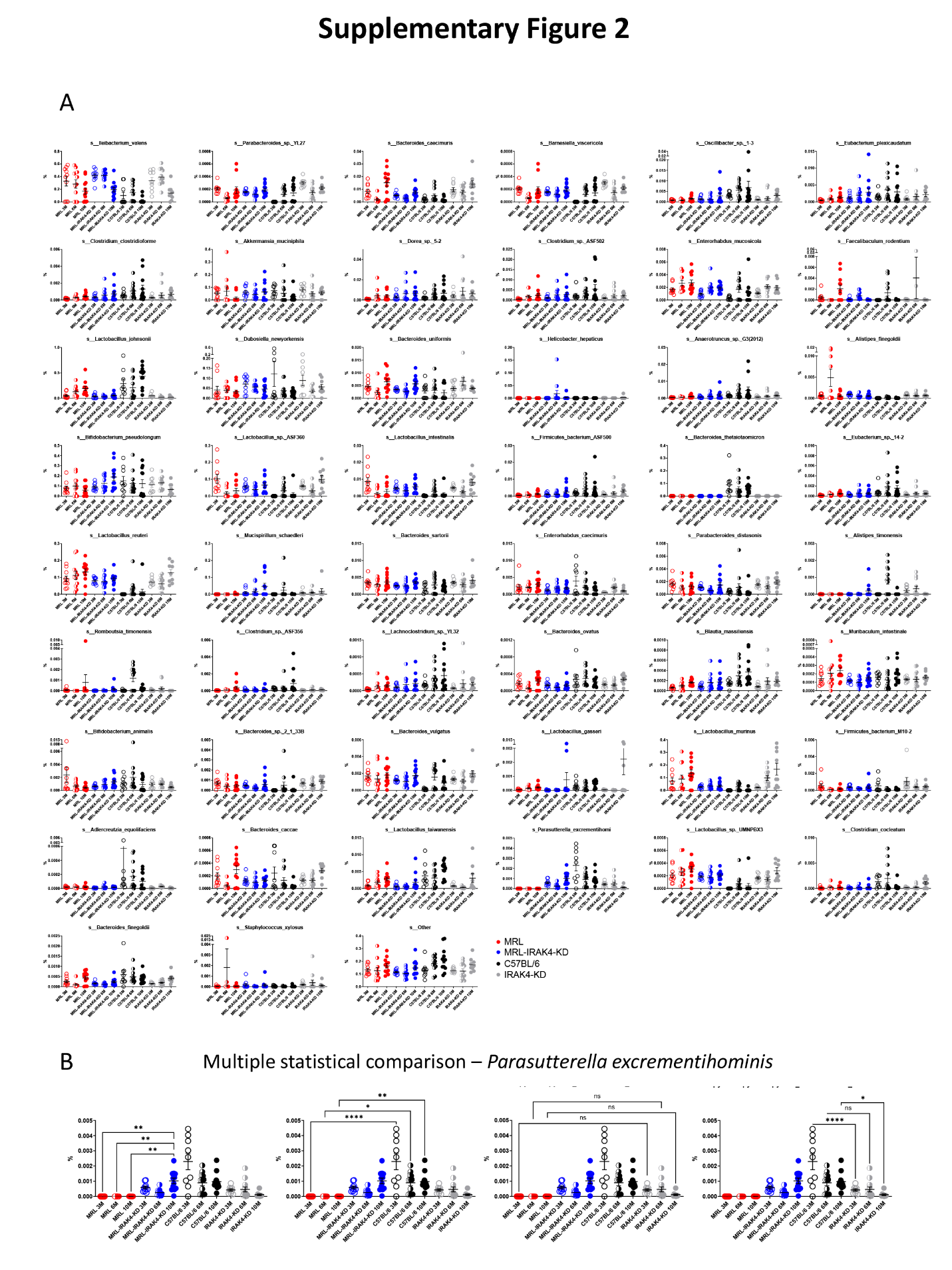

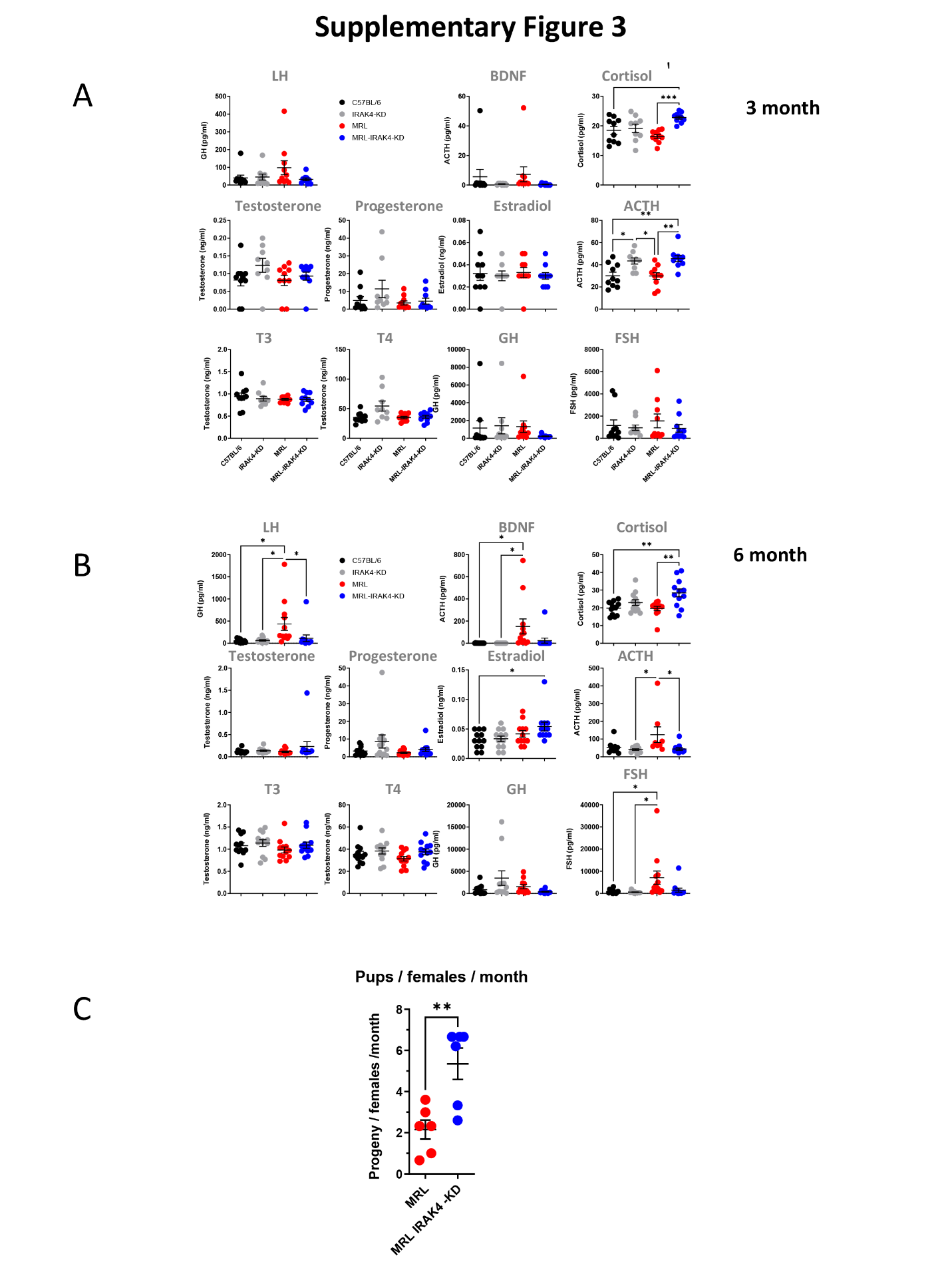

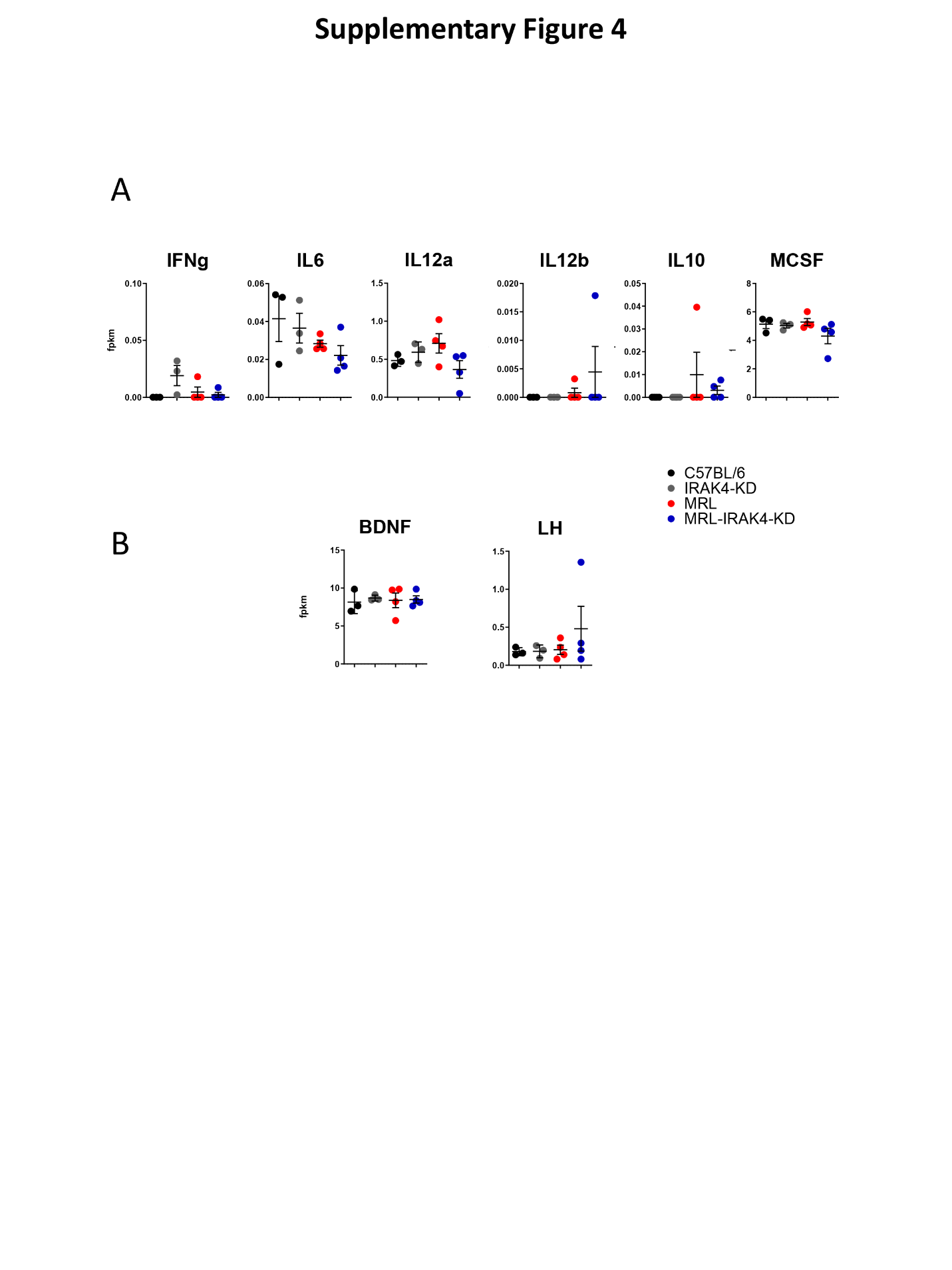
**

**
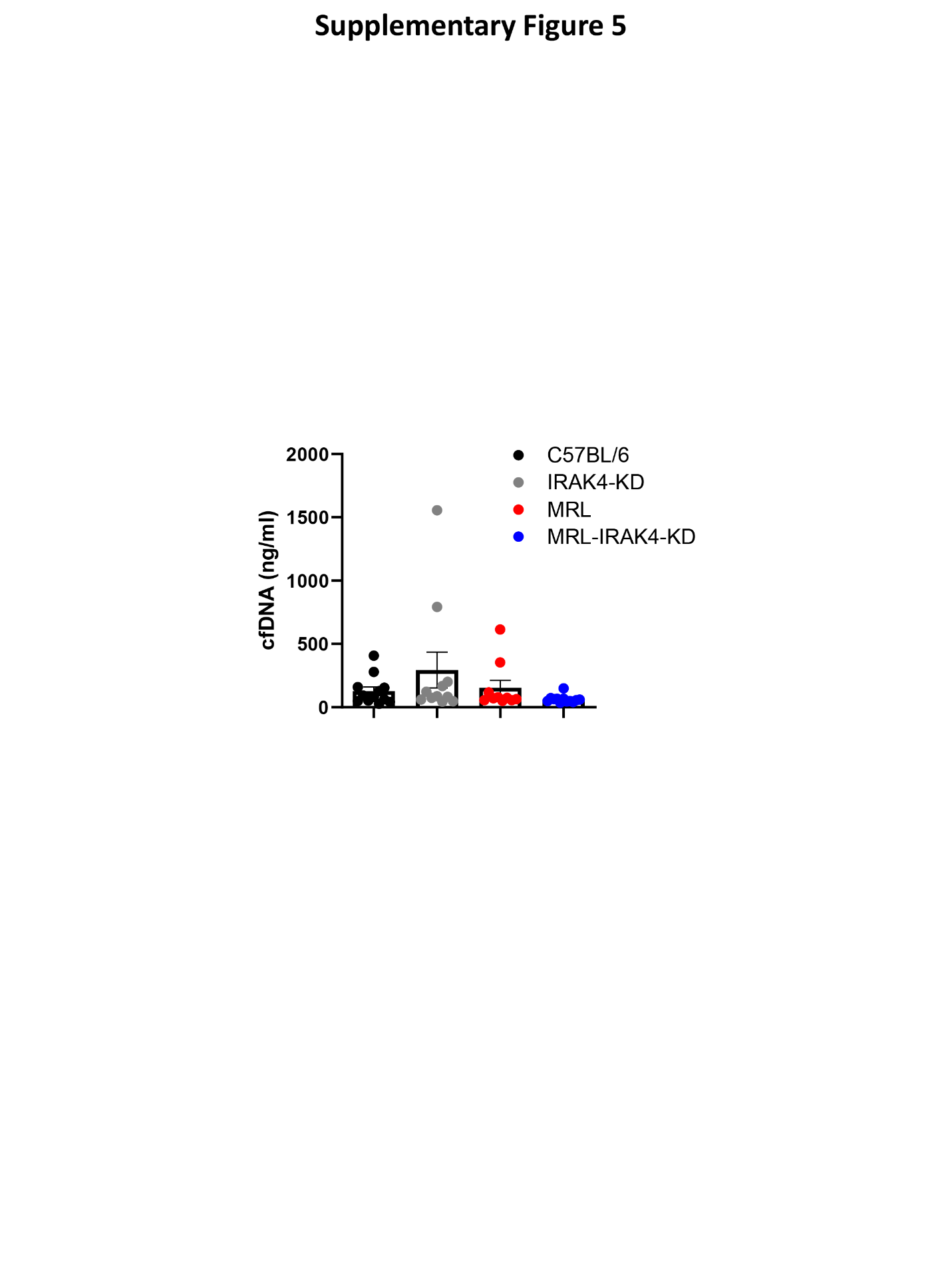
**

**
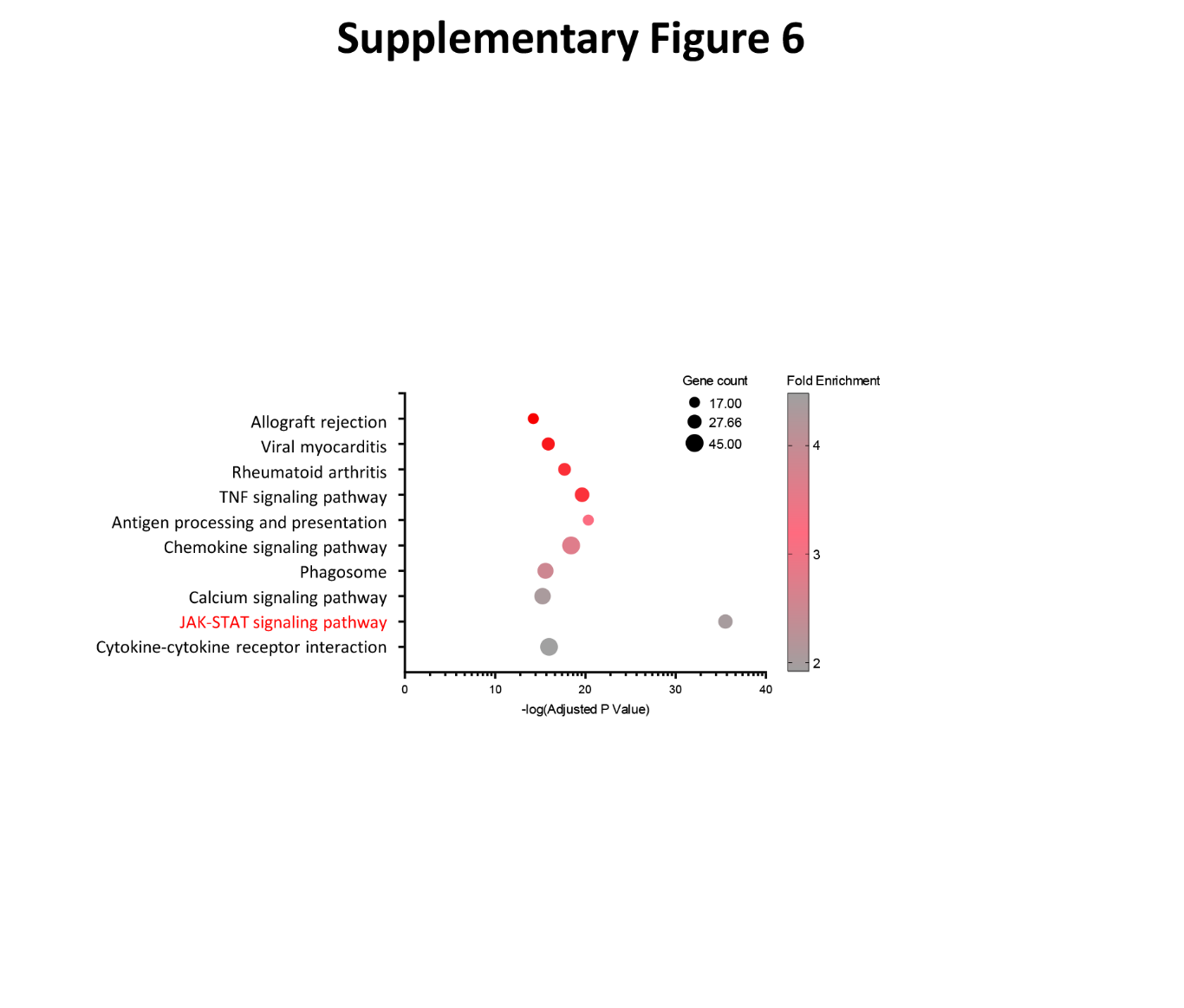
**

**Supplementary Methods**

**Microbiome analysis**: Mouse feces were collected, immediately frozen at -80°C and shipped to Diversigen (New Brighton, MN) where the 50 most abundant bacteria species were identified by RNAseq and frequency established.

### **Cell-free (Cf) DNA isolation and quantification:** DNA was extracted from mouse serum using DNA Extractor SP Kit (FUJIFILM Wako Chemicals U.S.A. Corp.), according to the manufacturer’s instructions. Next, cell-free DNA was quantified using the Quant-iT PicoGreen dsDNA Assay Kit (Invitrogen) according to the manufacturer’s instructions.
